# Determination of the effective anticandidal concentration of denture cleanser tablets on some denture base resins

**DOI:** 10.1590/1678-7757-2017-0077

**Published:** 2018-01-16

**Authors:** Yeliz Hayran, Işıl Sarikaya, Ali Aydin, Yadel Hazır Tekin

**Affiliations:** 1 Gaziosmanpaşa University, Faculty of Dentistry, Department of Prosthodontics, Tokat, Turkey; 2 Gaziosmanpasa University, Faculty of Science and Art, Department of Molecular Biology & Genetics, Tokat, Turkey

**Keywords:** Candida albicans, Surface properties, Denture cleansers, Denture bases

## Abstract

**Objective:**

Although the effectiveness of chemical cleansing against *Candida albicans* biofilm has been shown, the effective concentration of denture cleanser tablets has not been studied. The aim of this study was to assess the effect of three denture materials against *Candida albicans* biofilm and to determine effective concentrations of denture cleanser tablets.

**Material and methods:**

The surface-roughness of Acron-hi™, QC-20™ and Deflex™ (n=45 per resin) resins was standardized by using a profilometer and their contact angle or surface free energy was calculated. *C. albicans* biofilm was formed on all three resins and were treated with Polident 3 min™, Corega™ and Fittydent™ cleanser solutions at various concentrations and both resin-biofilm and cleanser-biofilm interest were determined by using a MTT protocol according to the European Committee on Antimicrobial Susceptibility Testing's antifungal susceptibility testing (AFST-EUCAST). Scanning electron microscopy was used to compare the efficacy of different resin materials against *C. albicans* biofilm. Anticandidal activity and surface free energy statistical parameters were calculated by using 3-way and 1-way ANOVA, respectively (p<0.05).

**Results:**

Polident 3 min™ and Corega™ tablets significantly inhibited (p<0.05) the proliferation of *C. albicans* against all denture resins at 27-37 mg/mL. Scanning electron microscopy results indicated that there was no significant difference among resin specimens regarding biofilm formation on dentures. We failed to find a significant relationship between surface free energy and the anticandidal effect of resin types. However, the polarity value of the resins was statistically associated with their anticandidal activity.

**Conclusions:**

The polarity of the resins, the concentrations of tablets and the chemical content of the cleanser may directly affect *C. albicans* biofilm formations. Polident 3 min™ and Corega™ tablets should be suggested for patients who use any denture resin types, whereas the Fittydent™ tablet should only be proposed for those who use Deflex™, when two tablets are dropped into 150 mL water.

## Introduction

Denture stomatitis is a common infection of the oral mucosa in denture wearers and *Candida albicans* is the most significant etiological agent of denture stomatitis^15^ ^,^ ^20^. *C. albicans* is an obstinate infection agent which is difficult to eliminate once it has been colonized as a complex biofilm formation^6^ ^,^ ^8^ ^,^ ^14^ ^,^ ^15^. The surface of acrylic resin denture base provides an ideal environment for microorganisms and biofilm formation, thus the development of *C. albicans* in such places^4^ ^,^ ^6^ ^,^ ^9^ ^,^ ^14^ ^,^ ^15^ ^,^ ^20^ ^,^ ^24^ ^,^ ^27^ ^,^ ^29^. The risk of denture stomatitis increases in the presence of poor oral and denture hygiene, misfit prosthesis and night wear of removable dentures^4^ ^,^ ^14^ ^,^ ^20^ ^,^ ^24^. It has been found that repeated inhalation and ingestion of microorganisms adhering to the mucosa and denture base can be a reason for various infections in patients with immune deficiency or in those receiving treatment^20^. Therefore, oral and denture hygiene is very important to remove microorganisms. Two methods are recommended to remove denture biofilm: mechanical or chemical, or a combination of both. Although the efficiency of mechanical methods in removing denture biofilm or microorganisms has been clearly shown, some people do not have the ability to apply sufficient denture hygiene^14^ ^,^ ^21^. This is especially the case for patients with limited motor capacity who have difficulty in cleaning the prosthesis with mechanical methods. To use unsuitable toothbrush with a dentifrice may also lead to surface roughness, which allows more microbial colonization^14^. The effectiveness of chemical cleansing to control *C. albicans* biofilm is shown in many studies, and denture cleansers are recommended for reducing biofilm formation on the dentures for these patients^4^ ^,^ ^6^. These cleansers are available as commercial products, and they usually include alkaline peroxides^19^ ^,^ ^23^, sodium hypochlorite^5^ ^,^ ^29^, acids^29^, enzymes^19^, and neutral enzymatic peroxides solution^4^ ^-^ ^6^ ^,^ ^15^ ^,^ ^19^. Effervescent tablets yielding an alkaline peroxide dilution with water are the preferred denture cleansers^3^ ^,^ ^7^ ^,^ ^13^ ^,^ ^16^ ^,^ ^18^ ^,^ ^22^ because they can easily provide enough cleansing without causing damage to surface resins^26^. These effervescent tablets act differently as mechanisms against microbial flora. For example, Polident 3 min™, one of the cleanser effervescent tablets, achieves chemical cleaning by using the release of oxygen from a neutral enzymatic peroxide solution^4^ ^-^ ^6^ ^,^ ^15^ ^,^ ^19^. However, the biofilm layer often cannot be completely removed from the resin surface and a number of viable cells remain on resins^29^. According to our knowledge, three significant factors – resin types with physicochemical features, cleanser types and cleanser concentrations – can be suggested to explain this situation.

PMMA, one of the resins, is the most commonly used denture base material due its favourable mechanical, physical and aesthetic properties. However, it has some disadvantages, such as low flexural and impact strength^10^. Therefore, alternative materials, such as polyamide thermoplastic resin and chemical modification of PMMA with high impact resin, have been developed to achieve better mechanical properties of denture base materials. High impact acrylic resin has a high resistance against unexpected falls^25^. Also, polyamide thermoplastic resin is more elastic than PMMA. Polyamide resins are especially preferred for patients with tissue allergies to PMMA^30^. Thus, polyamide thermoplastic resin and high impact resin are suggested for patients with a tendency to drop their prosthesis, such as elderly and handicapped denture wearers.

In addition, one of the significant physicochemical features of resin surface is the surface free energy (SFE) resulted from the asymmetry between the energies of the molecules at the surface and in the bulk of resin, since the molecules at the surface of a solid- phase material are under the pressure of a one-side force, whereas in the bulk material, molecules do not have net forces due to being under equal pressure from every direction. Surface free energy (SFE) and surface roughness (Ra) both have important roles in the first adhesion of microorganisms^2^ ^,^ ^17^. Some studies showed that Ra and, to a lesser extent, SFE of resins, along with environmental conditions, are responsible for the *C. albicans* biofilm formation on the resin surface^2^ ^,^ ^17^. However, the effect of the Ra on biofilm formations can be minimized and standardized by polishing resin surfaces to see the net effect from the SFE.

The effectiveness of various denture cleanser tablets in removing *C. albicans* biofilm formation on denture acrylic resin surfaces has been evaluated in other studies^4^ ^,^ ^6^ ^,^ ^15^ ^,^ ^29^. These studies showed a significant decrease in the amount of *C. albicans* after exposure to different cleansers^4^ ^,^ ^6^ ^,^ ^14^ ^,^ ^29^.

Cleanser concentrations may also play a significant role in the removal of *C. albicans* biofilm from resin surfaces. To the best of our knowledge, this is the first work that describes the correlation between cleanser concentration and biofilm removal from denture surface.

Thus, the purpose of this study was to evaluate the effect of different resin types on *C. albicans* biofilm formation and to determine the effective concentrations of commercial denture cleansing tablets to remove biofilm formation according to the manufacturers’ recommendation times. Null hypotheses were that 1) there is no significant difference between the amount of *C. albicans* inhibited by chemical cleanser tablets in MTT assay; 2) There is no significant difference between the amount of *C. albicans* bind to resin types in scanning electron microscope (SEM) analyser and MTT assay; 3) There is no significant interaction between the amount of *C. albicans* inhibited by resin types, tablet types and tablet concentrations in all assays; 4) There would be no differentiation among the concentration of chemical cleanser in decreasing *Candida* levels; and 5) The amount of *C. albicans* adhesion would not be associated with resin polarity.

## Material and methods

### Specimen preparation

Two types of heat-polymerized PMMA resin and one type of thermoplastic polyamide resin were used for the fabrication of specimens (n=45 *per* resin). All denture base specimens were prepared according to the manufacturers’ instructions. Circular wax pattern discs with dimensions of 10 mm in diameter and 2 mm in thickness were prepared using a stainless steel mould^5^ ^,^ ^6^. Wax discs were invested in denture flasks followed by a compression moulding technique for conventional heat-polymerized acrylic resin (Q-type) (QC-20, Dentsply, Addlestone, UK) and high-impact heat-polymerized acrylic resin (A-type) (Acron-hi, Kemdent, Swindon, UK); then, wax discs were invested in injection flasks followed by a rapid injection technique for polyamide thermoplastic resin (D-type) (Deflex classic SR, Buenos Ares, AR) and afterwards melted with boiling water. The heat-polymerized acrylic resins were then packed into the mould, and the metal flasks were placed in a boiler unit for polymerization. The infection flask and thermoplastic polyamide resin cartridge were placed in the device, and the resin was injected into the mould. All flasks were allowed to cool down for 2 h. All specimens were immersed in distilled water for 24 h for residual monomer release^28^. Following this, specimens were labelled on one surface. Respectively, one side of each specimen was ground wet with 600, 800 and 1,000 grit emery paper to standardize surface roughness, which was measured using a profilometer (Taylor Hobson, Surtronic 25, Leicester, UK). Evaluation length and range were calibrated at 1.25 mm and 100 μm, respectively. Three readings were made for each specimen, and a mean value was calculated. For all resins, surface roughness (Ra) was standardized at 0.3±0.02 μm. After surface roughness measurements were completed, the specimens were ultrasonically (Pro-Sonic 600, Sultan Healthcare, Hackensack, NJ) cleansed in sterilized distilled water at 50°C, at 28 kHz frequency for 10 min. Thus, any contaminants or artefacts from the surfaces were removed before the measurement of surface free energy (SFE).

### Contact angle and surface free energy measurements

For the contact angle and SFE calculation, three liquids with well-established polar and dispersive components of surface tension were chosen. Distilled water, diiodomethane and formamide were used with the sessile drop technique on the surface of the specimens for measurement. It is known that water and formamide are polar and that diiodomethane is nonpolar. Therefore, the water-formamide pair gave accurate values for polar components, and the water-formamide-diiodomethane combination gave better results in the calculations of both polar components and dispersive components. Dispersive, polar, acidic and basic components of the SFE and the SFE of denture resin surfaces were calculated using five different methods: the acid-base approach; equation of state; OWRK/Fowkes; Wu; and Zisman. Contact angle measurements were obtained using a KSV Attension Theta Lite Optical Tensiometer (Helsinki, FI). Five drops were measured on each sample at room temperature. SFE was calculated using contact angle values.

### Fungal cultures and growth conditions

The *C. albicans* (ATCC 1023) strain was maintained on solid Sabouraud dextrose agar at 35°C in an incubator. Colonial growth after 24 h was 1-5×10^6^ cells, measured with the help of trypan blue or a Neubauer chamber and pipetted of the polystyrene microtiter plates with 24 wells containing RPMI-2% glucose liquid medium and then allowed to grow at 35°C for 48 h in a shaker incubator at 150 rpm. The *C. albicans* cell counting with the Neubauer chamber was also standardized according to McFarland turbidity standards for experimental confirmation.

### Test tablets

**Figure 1:**
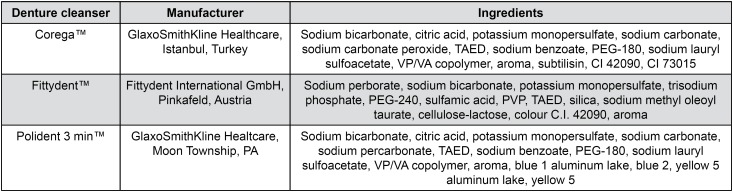
The chemical content of the Corega™, Fittydent™ and Polident 3 min™

Tablets of the alkaline peroxide denture cleansers Corega™ and Fittydent™ and the neutral peroxide enzymatic denture cleanser Polident 3 min™ were obtained from the manufacturers and used as received. The tablet contents (Figure 1) were reconstituted by using distilled water, and the cleanser working solution was used immediately. One test tablet is approximately 2.5 g. For cleanser working solutions, tablets were dissolved in a concentration ranging from 1-5 tablets/150 mL of warm distilled water, corresponding to 16, 32, 48, 64 and 80 mg/mL, respectively.

### Susceptibility testing

An MTT assay gives an accurate estimate of the number of viable cells. Thus, we performed an MTT assay according to AFST-EUCAST guidelines. An MTT stock solution (5 mg of MTT/mL of distilled water) was filter sterilized and kept at −20°C until use. First, the biofilm was grown as described previously. After a 48-h incubation period, the old medium in the wells was carefully removed, and the cells were treated with 200 μL of the cleanser solutions of Polident 3 min™, Corega™, and Fittydent™ for 3, 5, and 5 min, respectively, at final concentrations of 16, 32, 48, 64, and 80 mg/mL. Afterwards, cleanser solutions were replaced with fresh RPMI-2% glucose liquid medium containing MTT (final concentration, 0.5 mg/mL). The mixture was incubated for 4 h on a shaker incubator (150 rpm at 35°C). After the incubation period, 180 μL of the medium were removed, 30 μL of Sorenson's buffer and 150 μL of DMSO were added to the well, and the plate was vortexed for 5 min. The optical density of sample and blanks (DMSO with Sorenson's buffer) was measured with a spectrophotometer at 560 nm, with 690 nm as a reference interval. The percentage of viability was calculated using the Excel software. Each experiment was repeated at least three times for each of the cleanser tablets.

### Calculation of % inhibition and IC_50_

The half maximal inhibitory concentration (IC_50_) represents the required concentration of an agent to inhibit a biological process by 50% *in vitro.* The MTT assay results were reported as the percentages of viability of the test substances. The IC_50_ of the test compounds was calculated using these percentages of viability with the help of the XLfit5 software (IDBS) and expressed in μg/mL at 95% confidence intervals.

### Determination of the minimum inhibitory concentration

The minimum inhibitory concentration (MIC) of tablets was examined using *C. albicans* growing in the RPMI-2% glucose liquid medium. A 24-well microtiter plate was used for this measurement. Test compounds (16, 32, 48, 64, and 80 mg/mL) together with different resin types bearing *C. albicans* biofilm were incubated at 20-25°C for 3-5 min in air. Any plate well showing no visible growth was recorded as an MIC value.

### Scanning electron microscopy

Scanning electron microscopy (SEM) was conducted using biofilms of *C. albicans* formed on the surface of resins. Biofilms were treated with the test compounds with the IC_50_ concentrations for 3-5 min. Cells were then washed twice with DPBS and fixed in 2.5% glutaraldehyde in a phosphate buffer for 16 h and, shortly after, refixed in 2% osmium tetroxide for 2 h. Then, they were dehydrated through ethanol rinses (30, 50, 90, 95, and 100%) and mounted and sputter- coated with gold. Sample surfaces were examined using SEM (Zeiss LEO 440, Cambridge, UK).

### Statistical analysis

The statistical significance of differences was determined by the three-way analysis of variance (three-way ANOVA) followed by Tukey's test. Data that did not show homogeneity variance were analysed by the non-parametric Kruskal-Wallis test. The SPSS for Windows computer program was used for statistical analyses. Results of SFE were reported as mean values±SD of three independent assays, and differences among groups were considered to be significant at *p*<0.05.

## Results

**Figure 2:**
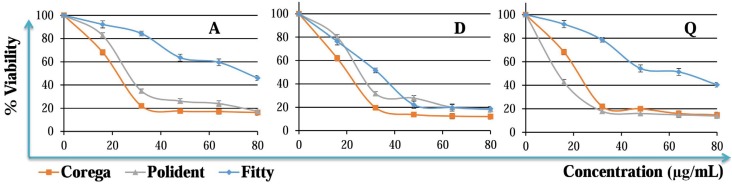
The effects of Corega, Polident 3 min or Fittydent tablets on the viability of *Candida albicans* biofilm on the surface A-, Q-, or D-type resin specimens. Exponentially growing cells on denture surface were incubated with Corega, Polident 3 min or Fittydent cleansing solutions for 5, 3, and 5 min, respectively, and the cell viability was measured by the MTT assay. Percent viability was reported as mean values±SEM of three independent assays (p<.05)

Anticandidal effects of the three cleansing tablets against *C. albicans* biofilm were initially screened using the MTT viability assay. According to MTT test results, Polident 3 min™ and Corega™ tablets exhibited strong anticandidal effects on *C. albicans* biofilm on all denture resin at nearly 2 tablets/150 mL of water concentration (*p*<0.05), whereas Fittydent™ had only anticandidal effects against the biofilm on D-type resin at nearly 2½ tablets/150 mL of water concentration (Figure 2) (Table 1). The anticandidal activity of the Corega™ was higher on A- and D-type resins compared to Polident™ and Fittydent™. The effective concentrations of Corega™ tablets were found to be 30.42 mg/mL, 27.37 mg/mL and 30.52 mg/mL (approximately 2 tablets) on the A-, D- and Q-type resins, respectively (Table 1A). However, Polident 3 min™ anticandidal activity was strongest on the Q-type denture (22.78 mg/mL, approximately 1½ tablet). The effective concentrations of Polident 3 min™ tablets were 37.62 mg/mL, 36.44 mg/mL (approximately 2½ tablets), and 22.78 mg/mL (approximately 1½ tablet) on the A-, D-, and Q-type resins, respectively. The effective concentrations of Fittydent™ were 74.89 mg/mL (approximately 4½ tablets), 38.19 mg/mL (approximately 2½ tablets) and 64.41 mg/mL (approximately 4 tablets) on A-, D-, and Q-type resins, respectively. IC_50_ values to be used in subsequent studies were determined by performing the MTT assay, as indicated in Table 1A.

**Table 1:** A) IC50 values for Corega, Polident 3 min or Fittydent cleansing tablets. B) MIC values for Corega, Polident 3 min or Fittydent cleansing tablets

A			
Inhibitor concentration (IC50 mg/mL)	A	D	Q
Corega	30.42	27.37	30.52
Polident 3 min	37.62	36.44	22.78
Fittydent	74.89	38.19	64.41
**B**			
Minimum inhibitory concentration (mg/mL)	A	D	Q
Corega	45	45	45
Polident 3 min	45	45	45
Fitty	80<	50	80<

After an incubation time, the inhibition zone (optically clear) was produced by each cleansing solution, and the lowest concentration at which there was no visible zone of inhibition was taken as the MIC. The experiment was repeated three times, and the MIC values are presented in Table 1B. As shown in Table 1B, Corega™ and Polident 3 min™ had a higher inhibitory effect against biofilm.

Statistical analysis was achieved by using a threeway ANOVA test and showed significant difference in the mean values of resin types, tablet types, and tablet concentrations. The three-way ANOVA was run on a sample of 135 resins to examine the effect of resin type, tablet type or tablet concentrations against biofilm. There was a statistically significant three-way interaction between resin type, tablet type and tablet concentrations, *F*(16, 90)=18.81, *p* = .000 (Table 2A). When calculating the two-way ANOVA, the resin types by tablet types (*F*(4, 90)=324.79, *p*=.000), resin types by tablet concentrations (*F*(8, 90)=16.56, *p*=.000), and tablet types by tablet concentrations (*F*(8, 90)=80.96, *p*=.000) are statistically significant (Table 2A). Application of Tukey's HSD multiple comparisons test showed a statistically significant difference among all test groups (Tables 2B and 2C) (mean difference is "*" indicating significant difference among groups).

**Table 2:** A) Tests of Between-Subjects Effects (Dependent Variable: Viability). B) Viability&Tukey HSD for resin types. C) Viability&Tukey HSD for cleansing tablets

A					
Source	Type III Sum of Squares	df	Mean Square	F	Sig.
Corrected Model	^a^88651.08	44	2014.80	408.41	.000
Intercept	180547.92	1	180547.92	36597.55	.000
Resin type	2953.44	2	1476.72	299.34	.000
Tablet type	22468.55	2	11234.27	2277.22	.000
Concentration	51486.42	4	12871.60	2609.11	.000
Resin type * Tablet type	6409.1	4	1602.27	324.79	.000
Resin type * Concentration	653.45	8	81.68	16.56	.000
Tablet type * Concentration	3195.23	8	399.4	80.96	.000
Resin type * Tablet type * Concentration	1484.90	16	92.81	18.81	.000
Error	444.00	90	4.93		
^a^R Squared = .995 (Adjusted R Squared = .993)					
**B**					
(I) Resin type	(J) Resin type	Mean Difference (I-J)	Std. Error	Sig.	
A	D	11,40*	0.468	.000	
	Q	6,69*	0.468	.000	
D	A	-11,40*	0.468	.000	
	Q	-4,71*	0.468	.000	
Q	A	-6,69*	0.468	.000	
	D	4,71*	0.468	.000	
					
**C**					
(I) Tablet type	(J) Tablet type	Mean Difference (I-J)	Std. Error	Sig.	
Corega	Polident	-4,31*	0.468	.000	
	Fitty	-29,27*	0.468	.000	
Polident	Corega	4,31*	0.468	.000	
	Fitty	-24,96*	0.468	.000	
Fitty	Corega	29,27*	0.468	.000	
	Polident	24,96*	0.468	.000	

**Figure 3:**
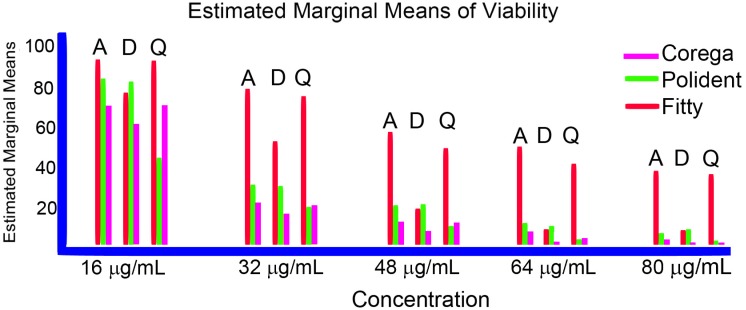
The graphics illustrated that an interaction effect is among resin*tablet at all concentration

The plot of the mean "viability" score for each combination of groups of "resins" and "tablets" are plotted in a line graph at all concentrations, as shown in Figure 3.

**Figure 4:**
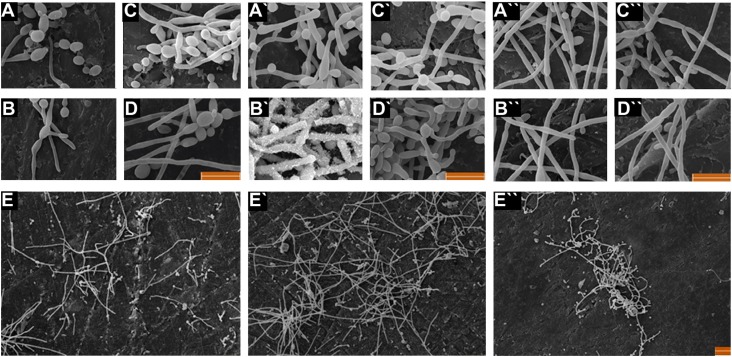
Scanning electron microscopy images of A, B, C, D, and E for A-type resin; A`, B`, C`, D` and E` for D type resin and A``, B``, C``, D`` and E`` for Q-type resin are Corega-tested surface, Polident-tested surface, Fittydent-tested surface, Control and Surface type, respectively. Bar is 10 μm. A- and Q-type resins showed more notched surface with deeply fissure (E and E``). A detailed analysis of the D (E`)-type resin surface displayed smooth and regular texture

The adhesion and the spreading of cells on surfaces were investigated using SEM. As shown in Figure 4, obvious cell spreading changes were not observed in the treated cells compared to the untreated cells. The biofilm exposed to the five concentrations of cleansing tablets did not exhibit significantly greater adhesion strength. This situation was not consistent with the results of the above mentioned MTT assays.

Also, we found that surface characteristics among the thermoplastic polyamide resin (D-type) and the PMMAs (Q-type and A-type) may cause the formation of the *C. albicans* biofilm layer in different confluences on resin types. In fact, we observed that the *C. albicans* biofilm layer effectively spread on the A- and Q-type resins by penetrating into their notched surfaces, whereas this was not found in the D-type resin (Figure 4, IV-VI).

Results of SFE analysis and the analysis of its components are shown in Tables 3A-3D. The dispersive and polar components of Q-, A-, and D-type resins were found to be 35.19-4.58 mJ/m^2^ for distilled water, 32.71-3.88 mJ/m^2^ for diodomethane, and 34.72-2.99 mJ/m^2^ for formamide. According to the acid-base approach, SFE values of Q-, A-, and D-type resins were 39.77, 36.60, and 37.71 mJ/m^2^, respectively. Q-type resin exhibited the highest SFE value (*p*<0.05), whereas the lowest values were found for A-type resin in all methods (*p*<0.05) (Table 3B and 3C). Generally, differences in the SFE of these resins were identified for all methods (*p*<0.05). Polarity values of Q-, A- and D-type resins were 0.13, 0.12, and 0.08, respectively.

**Table 3:** A) Acidic, Basic Components of Surface Free Energies of Test Liquids used in this work (mJ/m^2^). B) Surface Free Energy Components of PMMAs Surface Calculated by Acid-Base Approach (mJ/m^2^). C) Surface Free Energy of PMMAs Surface Calculated by the Other Methods (mJ/m^2^). D) Polarity and Average Contact Angle

A							
Liquid	γL	γLwL (γdL)	γ^AB^L (γpL)	γ+L	γ^–^L	Polarity (γ^p^L/γdL)	
Water (w)	72.8	21.8	51.00	25.5	25.5	2.34	
Diiodomethane (d)	50.8	50.8	0	0	0	0	
Formamide (f)	58.00	39.00	19.00	2.28	39.6	0.49	
**B**							
PMMAs	γL	γLwL (γdL)		γ^AB^L (γpL)		γ^+^L	γ^–^L
Q	39.77±0.76	35.19±0.76		4.58±0.48		1.03	2.28
A	36.60±1.28	32.71±2.56		3.88±1.74		0.99	1.94
D	37.71±1.45	34.72±0.89		2.99±1.12		0.79	2.14
**C**							
PMMAs	Equation of State	OWRK/Fowkes		Wu		Zisman	
Q	38.33±0.55	40.51±0.78		44.06±0.74		36.85±2.13	
A	35.81±0.90	37.39±1.09		40.95±0.98		32.27±8.98	
D	36.75±1.14	38.62±1.45		42.09±1.45		35.58±2.23	
**D**							
PMMAs	Polarity (γ^p^L/γdL)	θ° (Water)		θ° (Diiodomethane)		θ° (Formamide)	
Q	0.13±0.01	70.59±11.68		55.04±12.95		51.71±1.88	
A	0.12±0.06	81.93±5.09		52.72±4.52		56.81±1.88	
D	0.08±0.03	80.56±3.35		49.18±1.61		56.28±4.06	

According to the contact angle results, the wettability value of these resins was in the following order: Q-type (70.59) > D-type (80.56) > A-type (81.93) (Table 3D).

## Discussion

The null hypothesis that denture base material type, chemical cleanser type, different concentration of chemical cleanser solution and polarity of resin would not interfere with *C. albicans* biofilm growth was rejected.

First, we evaluated the anticandidal effect of two alkaline peroxide denture cleansers, Corega™ and Fittydent™, and one neutral enzymatic peroxide denture cleanser, Polident 3 min™, on three different resins so their surfaces were standardized to avoid surface imbalance. The SEM and surface analyses were used in this stage. It is known that polyamide resin surfaces generally exhibit a rougher texture compared to PMMA resins^6^ ^,^ ^12^ and the surface structure may lead to increased microbial flora and the attenuated effect of cleansers^1^ ^,^ ^24^. However, we utilised a simple process to obtain standardized surface roughness from three types of resin with different surface properties and applied a smoothing method that adjusted their roughness to 0.32±0.02 μm through a profilometer. However, even though a standardized surface roughness was used, all cells on the polyamide resin were very weakly attached to the surface and spontaneously separated more easily from the surface compared to PMMAs. These results showed that the surface properties of resins are not the only factor governing the *C. albicans* adhesion, and, at the same time, the chemical content of the material may affect the *C. albicans* adhesion (Table 1). That is why the Fittydent™ tablet had only anticandidal effects against biofilm on the D-type resin with the same administrative concentrations. In addition, the MTT analysis indicated that the polyamide resin with low polarity (Table 4D) exhibited a high anticandidal effect against *C. albicans* cells, whereas the PMMAs with high polarity had low anticandidal effect. The PMMAs, A-type resin, and Q-type resin exhibited approximately equal anticandidal effects, and one of the reasons may be that their polarity values were very close to one another. The bacterial attachment to resins has not been fully revealed to be affected by its SFE and wettability property because there are many inconsistent results from various studies^8^. For example, some studies showed a linear correlation between SFE values and *C. albicans* adhesion^2^ ^,^ ^17^, whereas other studies reported no correlation at al^9^ ^,^ ^27^. Likewise, in this study, we failed to find a strong correlation between SFE and *C. albicans* adhesion. It is speculated that low polarity, low SFE value and low wettability may lead to a significantly increased anticandidal effect. However, we found that merely the polarity feature of resins may alter its anticandidal affect. The SEM images substantially confirmed our speculations about resin types used in this study. For example, *C. albicans* biofilm layers on A- and Q-type resins were covered a much larger area and presented a higher level of growth than D-type resin. However, even though D-type resin showed rouger surface than the others, it can be pretty smooth and slippery following the surface deburring and polishing prosses. In addition, according to SEM presentations for all resins, we failed to find a significant difference between *C. albicans* forms such as yeast and hyphal formation.

Second, we conducted a MTT method to determine inhibitor concentration (IC_50_ mg/mL) and the minimum inhibitor concentration (MIC) values of these cleanser tablets. Results indicated that *C. albicans* viability was affected to alter the denture cleanser trademark, concentration, and resin type. Denture cleanser tablets act to biofilm layer in a concentration-dependent manner – that is, increasing the concentrations of denture cleanser tablets on biofilms layer lead to a gradual increase in the cell inhibition, showing a typical inhibitor effect. This means that the tablet concentrations are effective in terms of eliminating the biofilm layers. In addition, the concentration increasing effect reached maximum impact against cell viability at 64 mg/mL concentration (four 2.5 g effervescent tablets dissolved in 150 mL of water to prepare a 64 mg/mL solution). Regarding the efficacy of the tablets considered together with denture cleanser trademark and resin type, the Corega™ tablets should be advised to provide effective cleansing of A- and D-type resin (IC_50_, 30.42 and 27.37 mg/mL, respectively, correspond to approximately 2 tablets) and the Polident 3 min™ tablets are suggested for Q-type resin (IC_50_, 22.78 mg/mL, correspond to approximately 1½ tablet). However, Fitty™ tablets must be used in a more concentrated manner for the same effect on biofilm layer when compared withother types (IC_50_, 38.19 to 74.89 mg/mL, correspond to approximately 2½ to 4½ tablets). Other studies also showed that the type of resin of denture base affects the amount of *C. albicans* biofilm layers colonization, as observed in this study^4^ ^,^ ^6^. Murata, et al.^19^ (2010) reported that the influence of neutral enzymatic denture cleanser on the surface properties was less than that of alkaline peroxide denture cleanser due to the neutral enzymatic denture cleanser containing less peroxide. However, none of the denture cleanser tablet concentrations were able to remove *C. albicans* biofilm completely in up to 25 mg/mL concentrations (approximately 1½ tablet). Most studies were conducted to remove *C. albicans* biofilm formation on the denture base resins of PMMAs via denture cleanser tablets^15^ ^,^ ^29^, whereas, to the best of our knowledge, a few studies evaluated the efficacy of denture cleansers on thermoplastic polyamide resin^4^ ^,^ ^6^. One of the thermoplastic polyamide resin studies demonstrated smaller *C. albicans* growth on the PMMA surface than on the thermoplastic polyamide resin^4^. They found that the residual monomer was released from the PMMA, and they were putting this forward as a serious theory. Therefore, in this study, specimens were soaked in distilled water for 24 h after polymerization to reduce the residual monomer. The cytotoxic effects of the acrylic resins remained at high levels within the first 24 h following polymerization^28^. The water immersion method was suggested to reduce the level of residual monomer^11^ because this toxic effect is reduced in a time-dependent manner^29^. Another study determined that the cleanser tablets tested were more effective for PMMA resin than for thermoplastic polyamide resin^6^. This result was inconsistent with our findings. The reason we applied the surface roughness process to the resins using a profilometer was because of the varying study findings for both resins.

## Conclusion

We have clearly demonstrated that the polarity of resins and the chemical content of the cleanser may affect *C. albicans* biofilm adhesion. Also, the results clearly describe a high anticandidal effect that is directly dependent on the concentrations of tablets. Our finding suggested that the Polident 3 min™ and Corega™ tablets are suitable for patients who use any denture resin types, whereas the Fittydent™ tablet should only be advised for D-type resin users, and each cleanser solution should be prepared by two tablets and with 150 mL water. In summary, it was shown that anticandidal activity appears to be a function of the nature of the resins, their roughness, the type of cleanser and the specific concentrations of the cleanser.
